# Immune checkpoint receptors in regulating immune reactivity in rheumatic disease

**DOI:** 10.1186/s13075-014-0469-1

**Published:** 2014-10-29

**Authors:** Sabrina Ceeraz, Elizabeth C Nowak, Christopher M Burns, Randolph J Noelle

**Affiliations:** Department of Microbiology and Immunology, Norris Cotton Cancer Center, Geisel School of Medicine at Dartmouth, 1 Medical Center Drive, Lebanon, NH 03756 USA; Department of Medicine, Section of Rheumatology, Dartmouth-Hitchcock Medical Center, Geisel School of Medicine at Dartmouth, 1 Medical Center Drive, Lebanon, NH 03756 USA; Medical Research Council Centre of Transplantation, Guy’s Hospital, King’s College London, London, SE1 9RT UK; Department of Immune Regulation and Intervention, King’s College London, King’s Health Partners, London, SE1 9RT UK

## Abstract

Immune checkpoint regulators are critical modulators of the immune system, allowing the initiation of a productive immune response and preventing the onset of autoimmunity. Co-inhibitory and co-stimulatory immune checkpoint receptors are required for full T-cell activation and effector functions such as the production of cytokines. In autoimmune rheumatic diseases, impaired tolerance leads to the development of diseases such as rheumatoid arthritis, systemic lupus erythematosus, and Sjogren’s syndrome. Targeting the pathways of the inhibitory immune checkpoint molecules CD152 (cytotoxic T lymphocyte antigen-4) and CD279 (programmed death-1) in cancer shows robust anti-tumor responses and tumor regression. This observation suggests that, in autoimmune diseases, the converse strategy of engaging these molecules may alleviate inflammation owing to the success of abatacept (CD152-Ig) in rheumatoid arthritis patients. We review the preclinical and clinical developments in targeting immune checkpoint regulators in rheumatic disease.

## Introduction

Rheumatic diseases include inflammatory disorders that cause pain, inflammation, or damage in joints and other organs, resulting in significant morbidity, mortality, and societal costs. Examples that are felt to be the result of autoimmunity include rheumatoid arthritis (RA), juvenile idiopathic arthritis (JIA), systemic lupus erythematosus (SLE), psoriasis, systemic sclerosis (SSc), and Sjogren’s syndrome (SS). The magnitude of an inflammatory response is the net result of molecular pathways that enhance or temper immunity. Both genetic and environmental factors control these pathways and can influence the development and severity of these diseases. Beyond engagement of the innate immune system, the perpetuation and amplification of these pathologic processes requires signaling through the B-cell or T-cell receptor, followed by subsequent ligand interactions delivering co-stimulatory and/or co-inhibitory signals. These secondary signals are critical in determining cellular effector functions and modulating immunity to maintain homeostasis [1]. Co-stimulatory and co-inhibitory molecules belong to the B7/B7 ligand family and the tumor necrosis factor (TNF)/TNF receptor family. Their expression and functions are summarized in Tables 1 and 2.

**Table 1:** **B7/B7 ligand family members and functions**

**Molecule**	**Expression**	**Ligand/receptor**	**Function**
CD28	Resting T cells	CD80 or CD86 on APCs	Lowers TCR threshold
			Delivers a co-stimulatory signal
			Promotes T-cell proliferation, survival, cytokine production, T cell-dependent B cell functions [2]
CTLA-4/CD152	Resting/activating T cells	Outcompetes CD28 for CD80/CD86 binding	Increased TCR threshold
			Delivers a co-inhibitory signal [2]
			Upregulates indoleamine 2,3-dioxygenase
			CTLA-4^–/–^ mice develop autoimmunity [2]
PD-1/CD279	T cells, B cells, DCs, monocytes, NK T cells, exhausted cells and Tregs	PD-L1 on APCs, B cells, and T cells. PD-L2/CD273 on APCs. PD-L1/CD274 can also bind to CD80	Delivers an inhibitory signal
			Suppresses Bcl-xl [2]
ICOS/CD278	Activated T cells, T follicular helper cells	ICOSL/CD275 on APCs and B cells [2]	Induces proliferation
			Propagates germinal center reactions
			Upregulates IL-10 production [2]
BTLA/CD272	B cells, DCs, Th1 cells, macrophages	Herpes virus entry mediator [2]	BTLA delivers an inhibitory signal via ITIM and ITSM [2]
B7-H3/CD276	B cells, NK cells, T cells, activated monocytes [2]	Unknown	Co-inhibitory and co-stimulatory functions. Suppress antitumor responses [2]
B7-H4/B7S1/B7x/Vtcn1	APCs, cancer cells, and mRNA expression on nonhematopoietic tissue [2]	Unknown	Reduces cell proliferation and T cell interleukin-2 production [2]
B7-H6	Tumor cells [3]	NKp30	Binds NKp30 on NK cells resulting in interferon gamma and cytotoxic function [2]
VISTA/Dies1/Gi24/PD-1H	Highly expressed on murine myeloid cells. Low expression on T cells [2]	Unknown	Inhibits T-cell proliferation, reduces CD44 and CD69 expression [2]
			Increases cell motility [2]

APC, antigen presenting cell; BTLA, B and T lymphocyte attenuator; CTLA-4, cytotoxic T lymphocyte antigen-4; DC, dendritic cell; ICOS, inducible co-stimulator; ICOSL, inducible co-stimulator ligand; ITIM, immunoreceptor tyrosine-based inhibition motif; ITSM, immunoreceptor tyrosine-based switch motif; NK, natural killer; PD-1, programmed death-1; PD-L, programmed death ligand; TCR, T-cell receptor; Th, T helper; Treg, regulatory T cell.

**Table 2:** **Tumor necrosis factor/tumor necrosis factor receptor family members and functions**

**Molecule**	**Expression**	**Ligand/receptor**	**Function**
DR3	Lymphocytes	Tumor necrosis factor-like cytokine 1A (TL1A) expressed on APCs [4]	Delivers a co-inhibitory signal, induces cell survival, prevents apoptosis [4,5]
4-1BB/CD137	Activated T cells, Tregs, DCs and B cells	4-1BB ligand on DCs and B cells [6]	4-1BB induces CD8^+^ T-cell, NK T-cell and B-cell survival [6]
			Signaling back via 4-1BB ligand induces monocyte activation [6]
OX40/CD134	Activated T cells	OX40 ligand/CD252 on B cells, endothelial cells, DCs and macrophages	OX40 increases CD4^+^ T-cell survival/effector function [6]
			OX40 impacts immunoregulation by reducing interleukin-10 production by Tr1 and CD4^+^ Tregs [6]
CD27	Naïve T-cells, memory B cells, NK T cells, NK cells [6]	CD70 on activated lymphocytes and DCs	CD27–CD70 signaling on B cells propagates germinal center formation and plasma cell activities
			Signaling on T cells results in proliferation and cytokine production [6]
CD40	B cells	CD154/CD40 ligand on T cells, T follicular helper cells, endothelial and epithelial cells, B cells or APCs [7]	CD154^+^ T cells permit germinal center formation
			Signaling via CD40 on B cells induces B-cell differentiation, isotype switching and proliferation [7]
			Signaling via CD40 on APCs, increases CD80 and CD86 expression [7]

APC, antigen presenting cell; DC, dendritic cell; NK, natural killer; Treg, regulatory T cell.

The ability to interfere with the inhibitory function of checkpoint receptors CD152 (cytotoxic T lymphocyte antigen-4) and CD279 (programmed death-1) in oncology has proved successful. In 2011 the US Food and Drug Administration (FDA) approved ipilimumab, an αCD152 monoclonal antibody (Ab), for use in the clinic. In patients with metastatic melanoma, ipilimumab was found to effectively prolong survival and reduce metastases [8,9]. Manipulating the CD279 pathway has been shown to have remarkable efficacy in cancer patients. In patients with melanoma, nonsmall cell lung cancer, and renal cell carcinoma, treatment with BMS-936558, a CD279 blocking Ab, promoted anti-tumor responses [8]. Similarly, CT-011 (Cure Tech Ltd, Yavne, Israel), a humanized αCD279 IgG_1_ monoclonal Ab, safely induced remission in a subset of patients with hematologic malignancies [8]. In patients with solid cancers, tumor regression was noted following therapy with MDX-1106 (Medarex, Princeton, NJ, USA), an αCD279 IgG4 Ab, further demonstrating that the CD279 pathway plays a crucial role in cancer progression [2]. In addition to targeting CD279, there are ongoing phase 1 clinical trials investigating the role of CD279 ligands: CD274/programmed death ligand (PD-L)-1 in patients with solid tumors [ClinicalTrials.gov:NCT00729664] and CD273/PD-L2 in stage IV melanoma patients [ClinicalTrials.gov:NCT00658892]. The successes in manipulating CD152 and CD279/CD279 ligands in cancer provide proof of concept that targeting these molecules can have profound effects on the human immune response [8].

In contrast to the cancer studies, delivering an inhibitory signal or blocking a stimulatory signal to achieve endogenous immunosuppression is critical in autoimmune diseases. This was first shown in 2005 when the FDA approved the humanized fusion protein CD152-IgG_1_ (abatacept) as a treatment for RA [10]. The aim of this review is to discuss the function of co-stimulatory and co-inhibitory molecules in the pathogenesis of SLE, RA, JIA, SS, psoriasis, and SSc, as well as their potential use as therapeutic targets.

### Systemic lupus erythematosus

SLE is a chronic inflammatory disease targeting multiple organs including the skin, joints, kidneys, lungs, and central nervous system. During disease, autoantibodies to a spectrum of self-antigens, including nuclear antigens, develop and form immune complexes in various tissues. In the kidneys this complex formation results in glomerulonephritis (GN) [11-13]. Current therapies in SLE focus on both B-cell and T-cell targets. The αCD20 Ab rituximab (Genentech, South San Francisco, CA, USA) has been effective anecdotally, but failed to achieve significant benefit above background therapy in separate clinical trials of nonrenal and renal SLE [14,15]. However, it is now thought that these trials may have failed because the high dosage of glucocorticoids used could have masked the effect of rituximab [16,17]. In addition, drug efficacy is hindered by the lack of a definitive target antigen, impaired T-cell homeostasis by reduced CD4^+^ regulatory T-cell function, an altered T-cell repertoire that in turn promotes autoantibody synthesis [18], inflammation driven by T helper (Th)1 and Th2 responses [19], and soluble mediators such as interferon (IFN) alpha [20].

Positive co-stimulation through CD28 and negative regulation through CD152 are a major focus in designing immunotherapeutic agents to treat lupus patients. There is a wealth of evidence in murine models identifying interactions between CD28 and CD152 and their receptors, CD80 and CD86, as attractive clinical targets. In the spontaneous lupus-prone murine model NZBWF-1, where females develop fatal GN, CD152-Ig therapy effectively reduces autoantibody production [21]. In combination with cyclophosphamide, CD152-Ig effectively reverses nephritis and prolongs survival in NZBWF-1 mice [22]. Furthermore, prenephritic mice treated with CD152-Ig and αCD154 Ab have delayed disease onset and reduced anti-double-stranded DNA Abs [23]. Administered as a triple therapy combining CD152-Ig, cyclophosamide, and αCD154 Ab, prolonged remission and the presence of renal type II activated macrophages that serve as a biomarker of remission have been noted in this model [24]. In contrast to the success of CD152-Ig in murine studies, the humanized CD152-IgG_1_ fusion protein abatacept has been disappointing in the clinic due to an increase in serious adverse events found in a subset of patients in a phase IIb study [25]. A clinical trial investigating the efficacy of the combination of abatacept and cyclophosamide in human lupus nephritis is ongoing [ClinicalTrials.gov:NCT00774852].

There are several other observations that the CD28–CD152 axis is involved in the pathogenesis of lupus. Elevated levels of soluble CD28 are found in serum from lupus patients, which can inhibit T-cell proliferation *in vitro* [26]. Whether these levels are active in tempering disease is unknown. There is also an association between polymorphisms in the CD152 gene and SLE susceptibility in some ethnic groups [27]. How this polymorphism impacts disease progression is unclear because CD152 expression itself does not appear to be aberrant in SLE patients. However, studies have shown that CD152 may be functionally impaired in SLE, perhaps as a result of αCD152 autoantibodies [28]. Recently, one study has suggested that abatacept might be linked to regulatory T-cell repopulation [29]. Expression of CD152 ligands also appears to be relevant in SLE. High CD80 expression on CD4^+^ T cells correlates with disease severity [30], and treatment with αCD80 Ab reduces disease severity in the pristine-induced murine model of disease [31].

The CD278 (inducible co-stimulator)–CD275 (inducible co-stimulator ligand) co-stimulatory pathway may play a role in SLE pathogenesis. In SLE patients, CD278 is expressed on renal lymphocytes and peripheral blood T cells whereas CD275 is highly expressed on B cells but reduced on memory B cells, possibly due to recent interactions with CD278^+^ T cells [32,33]. *In vitro*, CD278 and CD3 stimulation leads to increased autoantibody synthesis from SLE peripheral blood mononuclear cells, indicating that CD278 contributes to disease and perturbed B-cell Ab responses [34]. This is further supported *in vivo* by reduced autoantibody production by CD278^–/–^ lupus-prone MRL/lpr mice [35]. In NZBWF-1 mice, prophylactic and therapeutic treatment with αCD275 Ab significantly reduced disease pathology [36], indicative that both the receptor and ligand are involved in perpetuating inflammation. The role of CD278 as a therapeutic target in human SLE is currently being evaluated in a phase Ib trial with AMG557, an αCD275 Ab [ClinicalTrials.gov:NCT00774943].

CD279 is an inhibitory receptor expressed on activated T cells that upon binding to CD274 (PD-L1) or CD273 (PD-L2) delivers a negative signal into the T cells [37]. In SLE patients, polymorphisms in the CD279 gene are associated with disease susceptibility [38]. In *vivo*, the role of CD279 is evident by the development of GN in CD279^–/–^ mice bred onto the lupus-prone strain lpr/lpr [39]. In NZBWF-1 mice, CD274 blockade increases CD4^+^CD279^+^ T cells and clinical pathology, demonstrating that blockade of the CD279:CD274 axis exacerbates disease [40]. In contrast, one study showed that αCD279 Ab treatment is associated with increases in CD8^+^ and CD4^+^ regulatory T cells and protection from disease in NZBWF-1 mice [41,42]. Because of these contradictory results, how the CD279 pathway signals in SLE remains unknown. One explanation is that the αCD274 Ab acted as a blocking Ab, which in turn would explain the increase in effector CD279^+^CD4^+^ T cells that produce IFNγ and perpetuate disease [40]. It is also possible that αCD279 Ab may delete CD279^+^ effector T cells, resulting in disease remission.

CD154 and its receptor, CD40, regulate both humoral and cellular immunity. CD154^+^ activated T cells can trigger B-cell activation, germinal center formation, long-lived Ab responses, and dendritic cell activation to facilitate the development of CD4^+^ and CD8^+^ T-cell responses [7]. Blocking this central co-stimulatory signal is an attractive target in lupus. An increase in CD154 expression on SLE lymphocytes correlates with disease severity in pediatric patients [43]. In active SLE, CD154 is highly expressed on lymphocytes and diminished in remission patients [44]. In a separate study, CD154 expression on T cells was found to induce CD80 on B cells, thus increasing their activation status and further propagating disease [45]. High levels of soluble CD154 have been detected in the serum of patients with advanced disease [46]. *In vivo*, αCD154 Ab therapy in NZBWF-1 mice prevents the onset of GN [47] and, when administered prior to the establishment of GN, diminishes renal immune complex deposition and prolongs survival [48]. Therapeutic intervention with αCD154 Ab in this disease model also reduces disease severity and resolves proteinuria [48].

In spite of the success of αCD154 Ab in murine models, the results of treatment in clinical trials are inconclusive [49]. In patients with active SLE, treatment with IDEC-131, a humanized αCD154 Ab, did not significantly improve disease scores or suppress anti-double-stranded DNA Ab or complement consumption [49]. However, this finding is confounded by the high placebo response in this trial due to the heterogeneity of SLE [49]. In a phase II trial in patients with proliferative lupus nephritis, BG9588 (Biogen, Inc., Cambridge, MA, USA), another αCD154 Ab, ameliorated disease activity, including proteinuria, and diminished CD38^+^ Ab-secreting cells [50]. Unfortunately, hematuria and thromboembolic events were reported in two subjects leading to concerns over the safety of targeting CD154 and to early termination of the trial [51]. Later studies have since reported that αCD154 Ab contributes to atherosclerosis and prothrombotic events [52]. This has prompted the reengineering of αCD154 Ab for safe use in SLE preclinical trials. Recently, researchers have shown that an αCD154 Ab containing a mutation in the IgG1 domain impairs Fc effector function and reduces thromboembolism in NZBWF-1 mice [53]. This observation suggests that further development of the αCD154 Abs with these modifications could increase their safety.

CD134 (OX40), a member of the TNF receptor superfamily, is a co-stimulatory molecule expressed on activated T cells and has been examined in SLE. CD134 expression on peripheral blood T cells from patients with lupus nephritis correlates with disease severity [30]. In the glomerular wall of lupus nephritis patients, CD134 expression on T cells and CD252 (OX40 ligand) expression on renal cells have been reported [54]. To understand the function of these molecules, *in vitro* assays have been performed to examine the function of both CD134 and CD252. For example, treatment of splenocytes from lupus-prone BXSB mice with αCD252 Ab, in combination with CD152-Ig, suppresses autoantibody production and proinflammatory cytokines [55]. Similarly, *in vitro* treatment of peripheral blood mononuclear cells from SLE patients with an αCD134 Ab reduces interleukin (IL)-4 and IL-10 and enhances IFNγ production whereas CD134-Ig reduces both Th1 and Th2 cytokines [56]. The method of targeting CD134 can exert different outcomes and warrants further investigation. For example, αCD134 Ab controls inflammation in lymph nodes while CD134-Ig prevented the onset of GN [56]. Collectively, these studies show that the CD134–CD252 pathway is involved in regulating inflammation by reducing the production of cytokines such as IL-4 and IL-10, known to perpetuate inflammation in SLE. To date, no clinical trials targeting the CD134 pathway have been conducted.

The CD70–CD27 and CD137 (4-1BB)–CD137 ligand (4-1BB ligand) co-stimulatory pathways belong to the TNF/TNF receptor family and signal on activated T cells. In SLE patients, impaired DNA methylation of CD70 on T cells is associated with disease progression [57] and expression of CD27 on memory SLE B cells and plasma cells correlates with disease severity [58,59]. At present, the CD70–CD27 pathway has yet to be extensively examined in murine lupus models. Several *in vivo* studies have investigated the role of CD137–CD137 ligand in SLE. CD137^–/–^ mice bred on a MRL/lpr background have increased autoantibody production, pathogenic T cells, and reduced survival [60]. Additionally, treatment of MRL/lpr mice with αCD137 Ab reduced CD4^+^ T cells, GN, and germinal center formation, as well as prolonging survival [61]. Similarly, αCD137 Ab therapy reduces disease severity in the NZBWF-1 model [62]. No clinical trials have been reported with either pathway in SLE.

### Rheumatoid arthritis

RA is a chronic systemic inflammatory disease characterized by destructive synovitis that, left undiagnosed, results in significant pain, deformity, and disability. RA predominately manifests in females and affects about 0.24% of the population [63]. Tissue inflammation and damage is mediated through several cell types, including T cells, B cells, monocytes, macrophages, and osteoclasts. Treatment of RA has been revolutionized by the use of biologics, including TNF inhibitors, rituximab, abatacept, and others beyond standard therapy, generally consisting of methotrexate.

One of the main pathways examined in RA involves CD28 and CD152 interactions with their binding partners, CD80 and CD86. Certain CD152 polymorphisms are associated with an increased risk of developing RA [64]. Soluble CD152 expression also correlates with disease severity, and membrane expression of CD152 on regulatory T cells is reduced [65,66]. Abatacept is effective in treating disease alone or in combination with methotrexate. Patients with low baseline levels of CD8^+^CD28^–^ T cells are more likely to achieve full remission when treated with abatacept [67]. Additionally, the frequency of CD4^+^CD28^–^ T cells decreases with treatment but it is uncertain whether this is a direct effect [68].

Other inhibitory B7 family members are also relevant in RA. CD279 polymorphisms are associated with increased susceptibility to disease, and soluble and membrane expression of CD279 is decreased in RA patients [69,70]. In mouse RA models, deficiency of CD279 or CD274 exacerbated disease [71,72]. Furthermore, treatment of mice with a CD274-Ig fusion protein marginally attenuates collagen-induced arthritis (CIA) [73]. High levels of soluble B7-H4, a B7 family inhibitory ligand, are associated with disease severity in RA patients as well as in murine models [74,75]. Both deficiency of B7-H4 and transgenically increased soluble expression of B7-H4 in CIA leads to accelerated disease, and treatment with a B7-H4-Ig fusion protein attenuates CIA [75]. AMP-110, a B7-H4-Ig fusion protein, is currently in a phase I study for use in RA [ClinicalTrials.gov:NTC01878123]. Co-inhibitory ligand CD272 (BTLA) and its receptor HVEM are found in affected synovium of RA patients [76,77]. Treatment of mice with HVEM-Ig increased disease severity in CIA [78]. No clinical trials involving BTLA and HVEM have been documented in RA.

The CD40 and CD154 pathway is implicated in RA pathogenesis. Some CD40 gene polymorphisms are associated with increased RA susceptibility. In addition, CD154 expression is increased in blood and synovial fluid T cells from RA patients [79,80]. IgM and IgG Abs against rheumatoid factor also correlate with increased levels of soluble CD154 [81]. Females with disease have greater expression of CD154 on CD4 T cells in comparison with healthy female controls, and the CD154 promoter is hypomethylated in females, but not males, with RA [82]. In CIA, αCD40 Ab treatment exacerbates disease, and αCD154 Ab and siRNA silencing of CD40 attenuates disease [83-86].

Other TNF superfamily checkpoint regulators are associated with RA. Soluble CD137 and CD137 ligand are also increased in RA patients in comparison with healthy controls, and αCD137 Ab treatment decreases CIA severity [87-89]. In addition, T cells expressing CD134 accumulate in effected synovial joints of RA patients. In CIA, both αCD134 Ab treatment and CD134-Ig treatment can attenuate disease [90-93]. This observation suggests that blocking engagement of CD134 with its ligand is responsible for this effect. CD70 is also overexpressed on CD4^+^CD28^–^ T cells from RA patients, and treatment of CIA with αCD70 Ab reduced autoantibody titers and disease severity [94,95]. Currently there are no clinical trials of these molecules in RA.

### Juvenile idiopathic arthritis

Immune checkpoint regulators are also implicated in the pathogenesis of JIA. Patients have an increased proportion of activated CD4^+^ and CD8^+^ T cells expressing CD25 and CD69, and lacking CD28 expression. There is a concomitant decrease in the naïve T-cell pool with shortened telomere length, suggestive of premature aging of this cell population [96]. In the CD8 T-cell compartment, CD28 negative cells have enhanced CD31 expression, allowing T cell receptor-independent activation of these cells [97]. In comparison with healthy controls, JIA patients have reduced numbers of dendritic cells in the blood and increased numbers in the synovial fluid. The dendritic cells in the synovial fluid express high levels of CD80, CD86, and CD40 in comparison with those in the blood, suggesting they are actively promoting inflammation in the joint [98]. Abatacept is FDA approved for the treatment of JIA alone and in combination with methotrexate on the basis of a double-blind withdrawal study [99]. Additional potential targets in this disease are B7-H4, identified as a susceptibility gene, and CD154, whose soluble levels are elevated in JIA [100,101].

### Sjogren’s syndrome

SS is an autoimmune systemic disorder that can occur by itself or in combination with other connective tissue diseases. Clinically, SS is characterized by dry eyes and dry mouth. This results from impaired exocrine function of the lacrimal and salivary glands, respectively, due to damage from a predominantly CD4^+^ T-cell infiltration [102]. The serologic hallmarks of SS include autoantibodies directed against ribonucleoprotein components SS-A (Ro) and/or SS-B (La) [103].

CD28 and CD152 interactions with CD80 and CD86 represent the best studied immune checkpoint regulators in SS. In SS patients, high levels of soluble CD28 are found in the serum and loss of expression on T cells is detected [26,104] and, *in vitro*, soluble CD28 inhibits T-cell proliferation in response to αCD3 [26]. This is further complimented *in vivo* in NFS/sld mice, a model of SS where CD4^+^CD28^–^ T cells in predisease mice express mRNA for IL-4, IL-10, and transforming growth factor beta, and prevent autoimmune lesion formation when adoptively transferred [105]. In SS, CD152 polymorphisms are linked to disease susceptibility and autoantibody production [106,107]. At the transcriptional level CD152 mRNA is found in salivary gland tissue, and αCD152 Abs are detected in the serum of SS patients suggesting the presence of this protein [28,108].

In addition to normal expression on antigen presenting cells, CD80 and CD86 are expressed on salivary gland epithelial cells and duct cells, and can be further upregulated in patients with sialoadentis with IFNγ [109,110]. On duct cells, CD80 and CD86 expression is associated with Th1 cytokines (IL-2 and IFNγ) and Th2 cytokines (IL-4 and IL-5), respectively [111]. This suggests that duct cells and other nonhematopoietic cells in the salivary glands express checkpoint regulator proteins that influence the development of inflammation. Therapeutically, NFS/sld mice treated with αCD86 Ab show reduced autoantibody production, T-cell activation, and a lack of autoimmune lesions, suggesting that the therapy eliminates CD28 co-stimulation [112]. In C57BL/6.NOD-Aec1Aec2 mice, another model for SS, treatment with an adeno-associated virus-2 vector encoding a CD152-Ig fusion protein reduced cell infiltration and Th1 and Th17 cytokine production [113], probably by binding to CD80 and CD86 and preventing co-stimulation through CD28. At present, a phase II study is currently recruiting inflammatory arthritis patients with SS to investigate the efficacy of abatacept [ClinicalTrials.gov:NCT02027298].

A few additional B7 family members have been examined in SS. For example, increased CD279 mRNA levels have been found in SS salivary gland tissue [108]. T cells in inflamed salivary glands express elevated levels of CD279, and CD274 expression on epithelial cells can be induced by IFNγ [114]. Since expression is intact, one speculation is that signaling through this co-inhibitory molecule pair is defective, because this potent negative checkpoint regulator pathway would be expected to control inflammatory T-cell responses in such a setting. One study has found in SS patients that salivary gland epithelial cells expressing CD275 can differentiate CD4^+^ T cells into T-follicular helper cells [115]. In one murine model, increased cell infiltration into the salivary glands has been reported in CD272^–/–^ mice [116]. These B7 molecules therefore play a role in determining the outcome of T-cell responses in SS and warrant further preclinical studies.

TNF superfamily members are also implicated in SS. Soluble CD154 is increased in the serum of SS patients and CD40:CD154 engagement can induce apoptosis of salivary gland cells [117,118]. However, treatment of nonobese diabetic mice with a vector containing CD40-Ig showed no significant reduction in inflammation in the salivary glands [119]. One study has linked CD252 polymorphisms with B-cell activation in patients with primary SS [120]. Similarly, the CD27–CD70 pathway is associated with B-cell abnormalities in SS. This association is demonstrated by the presence of CD27^+^ memory B cells in inflamed salivary glands [121,122] and overexpression of CD70 on CD4^+^ T cells due to hypomethylation of the CD70 promoter in SS patients [123].

### Psoriasis

Psoriasis is a chronic inflammatory skin disease characterized by hyperproliferation of kertinocytes, affecting approximately 2% of the population [124]. Some patients also have associated psoriatic arthritis, a destructive spondyloarthropathy. Treatments for psoriasis vary considerably depending on disease severity and the presence or absence of arthritis. These treatments include topical or systemic agents such as corticosteroids, retinoids, vitamin D analogs, ultraviolet light, methotrexate, cyclosporin, TNF inhibitors, and αIL-12/23 (p40) [125]. Little work has been done examining the role of co-stimulatory and co-inhibitory molecules during disease. However, studies with abatacept have shown that treatment can result in clinical improvement in patients with severe psoriasis and psoriatic arthritis [126-130]. In addition, elevated levels of CD154 are found on T cells from both psoriasis and psoriatic arthritis patients, suggesting that αCD154 Ab treatment may be effective in this disease [131,132].

### Systemic sclerosis

SSc is a complex autoimmune disease characterized by inflammation, vascular abnormalities, and fibrosis in the skin and internal organs and is more common in females than males. Early skin lesions demonstrate infiltration of activated CD4^+^ T cells, CD8^+^ T cells, monocytes, and macrophages [133-135]. In addition, autoantibodies are present that can directly promote fibrosis, and the pattern of antinuclear antibodies present can predict disease progression [136-138]. TNF superfamily co-stimulatory molecules have been associated with disease. Demethylation of CD154 regulatory elements in females is associated with CD154 overexpression on CD4^+^ T cells [139]. Patients also exhibit elevated plasma levels of soluble CD154. In bleomycin-induced skin sclerosis, a murine model of the disease, prophylactic αCD154 Ab treatment attenuated disease [140]. In addition, CD70 is overexpressed in T cells from patients with SSc and this correlates with demethylation of its promoter region [141]. Gene polymorphisms in CD134 are associated with increased susceptibility to SSc [142]. These observations suggest that therapies targeting CD154, CD70, and CD134 may be worth further investigation for the treatment of disease.

## Conclusions

**Figure 1:**
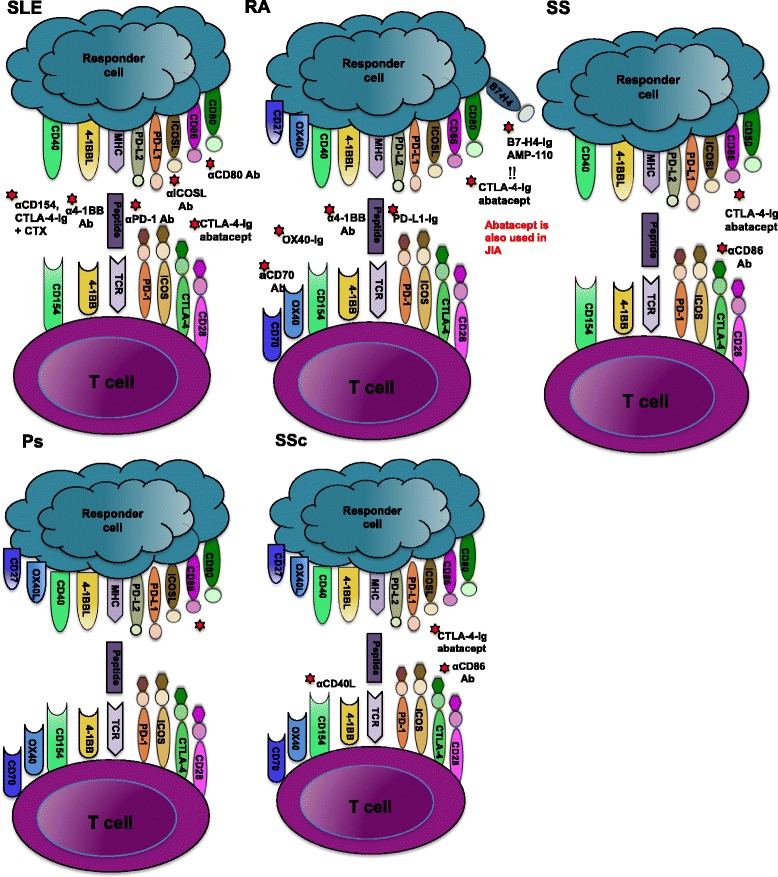
**Current** ***in vivo*** **immune checkpoint receptor therapies in rheumatic diseases.** T-cell activation requires two signals. The first is via the T-cell receptor (TCR), where peptide is presented by the major histocompatibility complex (MHC) on responder cells. The second involves a network of co-inhibitory and co-stimulatory molecules pathways such as CD80/CD86–CD28/cytotoxic T lymphocyte antigen-4 (CTLA-4), inducible co-stimulator (ICOS)–ICOS ligand (ICOSL), programmed death-1 (PD-1), programme death ligand-1/2 (PD-L1/PD-L2), 4-1BB–4-1BB ligand (4-1BBL), CD40–CD154 ligand, OX40–OX40 ligand and CD27–CD70. This diagram summarizes current therapies for manipulating these pathways to suppress disease in systemic lupus erythematosus (SLE), rheumatoid arthritis (RA), Sjogren’s syndrome (SS), psoriasis (Ps), and systemic sclerosis (SSc). Ab, antibody; CTX, cyclophosphamide; JIA, juvenile idiopathic arthritis.

In summary, checkpoint regulators represent viable immunotherapeutic targets for the treatment of both autoimmunity and cancer. A wealth of both mouse and human data indicate that co-stimulatory and co-inhibitory molecules are critical in a number of autoimmune rheumatic diseases (Figure 1). In addition, interventions in multiple murine models utilizing several of these pathways, either by blocking co-stimulatory receptors or by engaging inhibitory receptors, have profound therapeutic effects. However, abatacept is the only agent to achieve FDA approval in the autoimmune human diseases RA and JIA. To date, these findings have not translated into safe and effective therapy for other diseases. In light of the veritable explosion of effective treatments for RA and the approval of more agents for the treatment of psoriatic arthritis, a particular disappointment is the limited progress on this front in SLE. Expectations are still high that this will be achieved within the next few years. The current leading contenders for a successful agent in SLE from a checkpoint regulator perspective are αCD275, abatacept, or a modified αCD154 Ab. Perhaps SS and certainly SSc remain even more daunting disease targets. The intense ongoing investigation in this already fruitful area will undoubtedly produce more candidates for clinical trials in SLE and other rheumatic diseases in the near future.

## Abbreviations

Ab: Antibody
BTLA: B and T lymphocyte attenuator
CIA: Collagen-induced arthritis
FDA: US Food and Drug Administration
GN: Glomerulonephritis
HVEM: Herpes virus entry mediator
IFN: Interferon
Ig: Immunoglobulin, IL, interleukin
JIA: Juvenile idiopathic arthritis
PD-L: Programmed death ligand
RA: Rheumatoid arthritis
SLE: Systemic lupus erythematosus
SS: Sjogren’s syndrome
SSc: Systemic sclerosis
Th: T helper
TNF: Tumor necrosis factor
